# Hybrid Continuous Density Hmm-Based Ensemble Neural Networks for Sensor Fault Detection and Classification in Wireless Sensor Network

**DOI:** 10.3390/s20030745

**Published:** 2020-01-29

**Authors:** Malathy Emperuman, Srimathi Chandrasekaran

**Affiliations:** 1 School of Information Technology and Engineering, Vellore Institute of Technology, Vellore, Tamil Nadu 632014, India; e.malathy@vit.ac.in; 2 School of Computing Science and Engineering, Vellore Institute of Technology, Vellore, Tamil Nadu 632014, India

**Keywords:** wireless sensor network, sensor faults, neural networks, classification of faults, hidden Markov model

## Abstract

Sensor devices in wireless sensor networks are vulnerable to faults during their operation in unmonitored and hazardous environments. Though various methods have been proposed by researchers to detect sensor faults, only very few research studies have reported on capturing the dynamics of the inherent states in sensor data during fault occurrence. The continuous density hidden Markov model (CDHMM) is proposed in this research to determine the dynamics of the state transitions due to fault occurrence, while neural networks are utilized to classify the faults based on the state transition probability density generated by the CDHMM. Therefore, this paper focuses on the fault detection and classification using the hybridization of CDHMM and various neural networks (NNs), namely the learning vector quantization, probabilistic neural network, adaptive probabilistic neural network, and radial basis function. The hybrid models of each NN are used for the classification of sensor faults, namely bias, drift, random, and spike. The proposed methods are evaluated using four performance metrics which includes detection accuracy, false positive rate, F1-score, and the Matthews correlation coefficient. The simulation results show that the learning vector quantization NN classifier outperforms the detection accuracy rate when compared to the other classifiers. In addition, an ensemble NN framework based on the hybrid CDHMM classifier is built with majority voting scheme for decision making and classification. The results of the hybrid CDHMM ensemble classifiers clearly indicates the efficacy of the proposed scheme in capturing the dynamics of change of statesm which is the vital aspect in determining rapidly-evolving instant faults that occur in wireless sensor networks.

## 1. Introduction

Wireless sensor networks (WSN) are widely deployed in various applications such as environmental monitoring, transportation, health care and industrial automation [1]. These applications utilize large numbers of wireless sensor nodes to measure the parameters of interest with energy and computational constraints [2]. Recent sensor networks have the capability to handle huge data like image or visual sensing, which is in turn integrated with the cloud to enhance the capability of the internet of things (IOT) [3]. Furthermore, the WSN has been implemented in mission critical applications like body sensor networks, security, space, underwater communication, and military [4,5]. Often, critical sensor networks are installed in harsh, inaccessible, and uncontrolled environments, which may affect the sensor performance and cannot be attended immediately. Normally, sensors get affected due to the surrounding environment and various malfunctions within the sensor nodes [6,7]. Hence, faults in sensor nodes can create substantial loss of network failures and may even affect the economy of the concerned application [8]. Furthermore, fault data also affects the bandwidth and energy consumption of the network. Therefore, sensor fault detection is an important process, and it is essential for all wireless sensor networks [9]. Fault detection and diagnosis can improve the reliability of sensor networks and enhance its bandwidth of operation. There are various techniques as reported in recent research surveys related to fault detection and diagnosis in sensor networks [10,11,12,13]. Incidentally, it is worth noting that while choosing fault detection methods, care should be taken to reduce the energy consumption and ease of integration with the existing networks. On the other hand, the quality of service of the fault detection should not be compromised to ensure the effective detection of faults. Upon successful detection or diagnosis of faults, the node can be removed or inactivated. Hence, the data from other nodes are re-routed with alternative nodes in order to sustain the network life.

Depending on the appropriate application and domain of sensor network system, each sensor node can contain single or multiple sensors with supporting processor and wireless communication systems [14]. Faults may occur either in the hardware or software of a node, and they are classified as hard and soft faults. The hard faults represent the complete damage of hardware of the sensor whereby the component/device is unable to communicate with other devices. In soft faults, on the other hand, the node is in active mode but communicates erroneous readings to the central or neighboring nodes. Often, the node get damaged at various levels and stages such as during sensing, processing, storing, or actuation. The general sources of failure may be attributed to calibration error, hardware failure, hostile environment, very low battery, and link failure [8]. Based on the timing, faults are divided into three major categories, namely permanent, intermittent, and transient. Among these, transient faults are difficult to detect since the occurrence is only for a brief period i.e., on the millisecond to nanosecond scale. Transient faults are further subdivided as random faults and spike faults. These faults are particularly important in mission critical sensor networks. As an example, in the body sensor network, the data from the sensors that are installed internally and externally to the human body are very critical in deciding the health condition of the concerned human being [15]. Therefore, it is evident that the detection of faults in these types of the sensor networks with high accuracy is currently very important.

**Figure 1:**
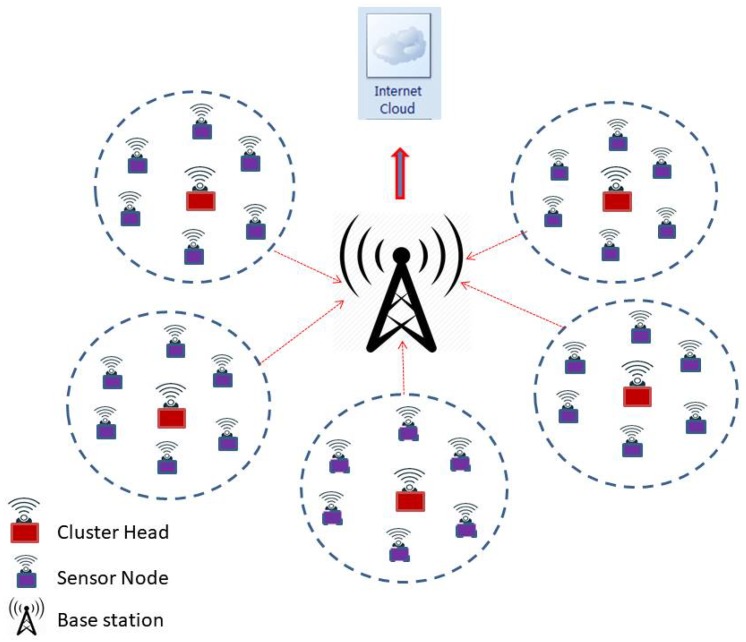
A typical wireless sensor networks (WSN) uses clustering technique for data communication from nodes to cloud.

A typical WSN that uses clustering-based data communication with the base station is shown in Figure 1 and the same structure of the network is taken up for research in this study and analysis. Here, we propose the continuous density hidden Markov model (CDHMM), which is well studied in several mechanical and electrical applications. However, the research work based on CDHMM in WSN has not yet been studied in detail for fault detection since there was no requirement. Recently, in the critical wireless sensor network and more specifically in health monitoring systems, such implementations have started receiving specific focus and attention from the research community under the WSN category. For these networks, very low level faults and more so transient responses are required to be detected at the nascent stages itself to ensure the reliability of network. Hence, this proposed works aims at assessing the dynamics of the sensor fault based on the optimized hidden state transitions, which is in fact reflective of the inherent changing dynamics of the faults in the sensors. In addition, hence, a comprehensive diagnostic system has been taken up in this study which utilizes a hybrid CDHMM-Neural Network implementation wherein the dynamics of the fault is obtained from HMM while the classification of the faults is carried out by ensemble NN systems.

### 1.1. Objective and Problem Statement

The main objective of the paper is to detect the permanent, intermittent, and transient sensor faults with better accuracy, as compared to existing techniques which suffer from the limitation of poor recognition due to the dynamic nature of faults. Therefore, a hybrid CDHMM with an ensemble of NNs, namely learning vector quantization (LVQ2), probabilistic neural network (PNN), adaptive probabilistic neural network (APNN) and radial basis function neural network (RBF) to detect and classify various faults in sensor node has been implemented. Further, to arrive at a credible and unbiased decision on the category of faults, a majority voting scheme is proposed.

### 1.2. Contributions

Unique contributions in this research study are summarized as follows:

1. Hybridization of continuous density HMM with NNs is the first of its kind proposed for the detection and classification of sensor faults in a WSN.
2. CDHMM is specifically used to determine the state transition of sensor data during faults. Based on the detection of such transitions, classification of faults and percentage of fault data, even the prediction of a sensor lifetime can be carried out more precisely.
3. When CDHMM is combined with NNs, the classification efficiency can be increased; even a low level of fault that occurs in the sensor data can exhibit a considerable change in the state transition. Besides, this can also detect the mixture of faults for different percentages of fault rates.

The second section focuses on the related works on sensor faults in WSN, with a detail discussion on the limitations in each technique. The third section introduces the continuous density HMM followed by various neural networks. The fourth section proposes the framework for fault detection and classification of different sensor faults. The fifth section discusses the results and analysis of various algorithms for different types of faults with varying fault rate percentage.

## 2. Related Works

Fault detection techniques have been first proposed for identifying machine and control system faults. The various faults that are studied in the machines are bias, drift, gain, precision degradation, complete failure, and constant output with noise. These faults are entirely different from those of faults that occur in the wireless sensor network [16]. Over the past few decades, artificial neural networks, support vector machine and k-nearest neighbor have been used for fault detection in machine applications [17]. These techniques have been successfully implemented for rolling fault detection and other machine control applications. However, it is evident that sensor fault detection necessitates complex techniques which are capable of including the dynamics of the network. Thus, the fault detection techniques which have been used to detect faults in machines are modified according to the requirement in sensor networks and implemented for the diagnosis of faults in the WSN.

The two important categories of faults in WSN are data centric and system faults. Data centric faults include spike, outlier, stuck-at, high noise, or variance. On the other hand, system view faults are namely calibration, connection or hardware, low battery, environment out of range, and clipping. These faults are elaborately discussed along with fault duration, pattern, and their impact on the sensor data [8]. Fault detection in WSN is basically classified as centralized, distributed, and hybrid approaches [18]. In the centralized approach, a computationally powerful node with large memory capacity with uninterrupted power supply is used for the fault detection and classification in the network. The implementation of this approach is complex but the accuracy of the detection is high when compared to distributed and hybrid approaches. In the centralized approach, [19] a proposed machine learning technique like the naive Bayes framework is used to find out the end to end transmission delay to determine the hardware faults in sensor nodes. Likewise, gradient based forwarding protocol is proposed to determine the node failures due to high data rates [20]. Similarly, Support Vector Machine (SVM) learning is reported in distributed learning in WSN and it has demonstrated the capability to exhibit better performance while scaling the network [21]. The SVM technique has also been used in the distributed technique for the classification and detection of sensor faults [22]. The SVM is also observed to adapt based on various kernel functions for nonlinear classification. SVM has been used to define a decision function and implement in the cluster head to detect the fault sensor. This approach has been used only to classify the fault or non-fault sensorm, not the types or dynamics of the fault.

In [23], authors proposed a simple technique to detect the fault sensor. Here, a threshold value was used to distinguish between the false and true data. This approach cannot be utilized for mission critical network, since this simple method may result in misclassification for low level of faults. In [24], used a belief function-based decision fusion method for detecting faults in the WSN. Four classification techniques are proposed to enhance the performance of the belief function fusion approach. This method cannot capture the dynamics of the individual sensor faults. In [25], a distributed algorithm was proposed that considered response time and detection accuracy in fault detection in addition to the required quality of service (QOS). The Bayesian model for classification and Pareto optimization to attain the required QOS was studied. The reduced data accuracy with this method restricted the use in critical sensor networks. Authors in [26], proposed an automated fault detection method using feed forward NNs which were trained by the hybrid Meta heuristic algorithm. This model was not suitable for instant or transient fault diagnosis since the fault state transition could not be captured with the NNs alone. In [27], used a semi-supervised method for the fault detection in WSN using pattern classification. As an estimator, posterior probability was used to find the normal or faulty nodes. Both online and offline fault detection could be performed with the proposed method. However, data accuracy with this approach may not meet the requirements of the critical sensor network.

In continuation to the distributed approach, [28] proposed the Neyman–Pearson test to detect the faults such as random faults, stuck at non-zero, stuck at one and stuck at zero in the sensor nodes. The faults of a sensor can be detected based on the neighboring sensor node data. Thus, the accuracy of the fault detection depends on the characteristics of neighbor nodes. Therefore, this technique results in error if the neighbor nodes get affected by faults. In [29], very simple statistical methods such as the Kuiper test and the autoregressive model are used for detection of sensor health. These statistical methods require a larger number of neighboring nodes data to determine the faulty sensor node. Likewise, [30] proposed the directional diagnosis method to detect the faults in the network or node. The detection was done with high accuracy by repeatable information probing and reasoning of fault. The authors in [31,32,33], proposed probability-based fault detection approach which used the transmission time of the packet from the sensor node, the incremental probe scheme, and the probabilistic inference model. Data aggregation used the Markov chain controller model, and pattern classification used a neighbor hidden conditional random field method to detect the fault sensor node. These methods, however, could not find the number of faults and types of faults in the network.

In [34], proposed a novel method for fault detection using a sequence-based detection method. They used the fletcher checksum algorithm and path planning to decrease the distance taken by the data to reach its destination. The network failure could be determined using the continuous path changes and also by using control messages to find the health of the concerned node. Further, comparison-based approaches have been proposed by [35,36], which pertain to the distributed fault detection method. This is implemented by neighbor sensor correlation, and reactive fault detection using temporal and spatial correlation. They do not function when the node starts to move. These approaches are primarily based on the neighbor node data, and if the neighbor sensor moves away from the range, then the detection gives an error reading.

In [37], fault sensor data detection is done in the cloud using clustering of WSN which increases the fast detection of error in sensor data and improves the detection accuracy. This approach is scalable to any topology of networks, and the detection is focussed within the spatial and temporal data sets. Therefore, the time it takes for detection of any amount of error can be drastically reduced. In [38], the detection of a sensor fault is done by the distributed approach with clustering of nodes. Determination of sensor fault is done by verifying the table associated with the neighbor sensor nodes. Also, the false alarms are mitigated by using time redundancy and therefore the instant false does not create a false alarm. The cluster-based method depends more on the efficiency of the cluster formation.

The soft computing method proposed by [39] reported the recurrent neural network to detect the faults using the neighbor’s data. [40] used principal component analysis with the neural network to determine the soft fault. [41] reported the fault detection using fuzzy inference system with its neighbor to detect the transient and permanent faults. These methods have drawbacks when the nodes are dynamic, resulting in inaccurate low level fault detection, and increased scalability of the network.

The centralized approaches have good detection accuracy compared to the distributed technique, but the implementation of the centralized system needs more computation power. In order to utilize both merits, a hybrid approach is proposed where the algorithm is implemented both in the cluster node and in each sensor node. In [42], the authors reported a hybrid distributed fault detection method using a mobile sink. It detects the fault in both the hardware and software of the sensor network. The detection of fault is accomplished by the cluster head, and it updates the status of each node in the network. This information is further used by the administrator to find out the fault in the sensor node. The main drawback of this method is the delay in detection due to the error in the path planning that determines the performance of the algorithm. The above-mentioned limitations can be overcome by proposing a hybrid method where the state transition of the fault data is captured by the hidden Markov model and the classification is carried out by neural networks.

## 3. Proposed Approach

In this section, we propose a centralized approach, where the HMM is hybridized with NNs, and is implemented in each cluster head of the networked sensors as shown in Figure 1. Although the energy and computation requirement for the proposed method is high, the requirement of low level fault detection in the critical sensor network is indispensable. Further, the recent sensor nodes are computationally powerful with less energy consumption. In this work, we detect only the individual sensor faults rather than the network faults. However, the same proposed method can be used for network fault detection using time correlation (inbuilt in the HMM) and space correlation which can be easily implemented using any one of the methods listed in [43]. Once the fault is detected, the data can be loaded onto the cloud or server for further processing and intimation. Thus, the proposed hybridized HMM with NNs is practically viable, and can be implemented in the online detection of faults in the networks which is the ultimate requirement for implementation in a real time environment. The rationale behind the formulation and implementation of hybridized NNs with CDHMM is attributed to its ability to correlate parameters related to the dynamics of state changes, its capability to associate with the effects of memory propagation during state transitions, its capacity to associate sequence similarity relationships, and its inherent capability to predict time-series sequences. These features are obviously necessitated in order to capture the transient and intermittent fault occurrences in wireless sensor nodes. In addition, the dynamics of the fault characteristics exactly matches with the proposed hybrid models. Thus, the preceding merits succinctly justify the selection of CDHMM and augur well for its suitability in implementing with specifically chosen NNs for the detection of faults in a sensor node.

### 3.1. Hidden Markov Model

**Figure 2:**
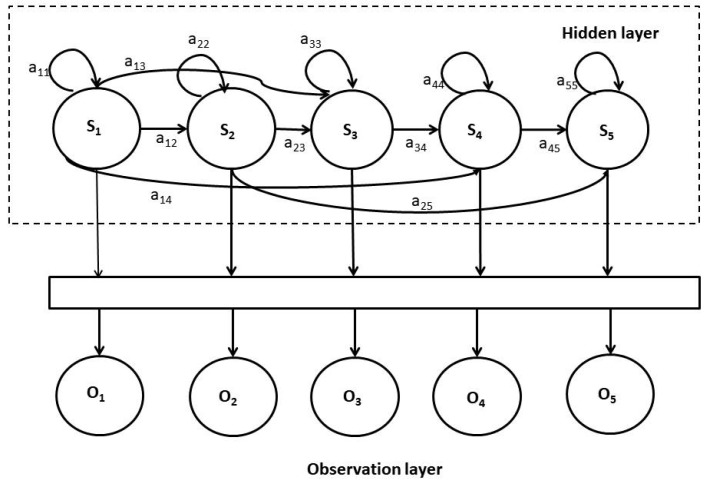
A five-State hidden Markov model (HMM) process.

In this research, a five-state HMM is adopted and the same is shown in Figure 2, where the top layer represents a Markov process which is the hidden process, while the bottom layer shows the observable states that depends on the hidden states. In this case, the hidden five states S = $S_{1},S_{2},S_{3},S_{4},S_{5}$ are connected with the output observable states as O = $O_{1},O_{2},O_{3},O_{4},O_{5}$ using transition probability matrix ‘*A*’. HMM is best described based on four parameters, namely the hidden states, initial, transition, and emission probability. If the observation is continuous in nature, then the continuous density HMM is formulated as λ=(A,B,Π), where Π and ‘B’ are the initial and the emission probability distribution respectively. Incidentally, it is worth mentioning that the appropriate choice of the type of HMM at the nascent stage and during the design phase of HMM formulation depends on the complexity of the problem, signal processing requirements and applications [44].

The procedure adopted during the implementation of continuous density HMM is indicated in Algorithm 2. In step 3, a minimum distance algorithm is used to cluster the data for training the dataset. The algorithm is primarily a minimum distance metric strategy (Euclidean), utilized to determine the adjacent datasets relative to various junction of intra-cluster and inter-cluster dataset. This is used to realize an optimum common crossing vector that act as the centers denoting the dataset. The various steps followed in the implementation of the minimum distance algorithm is shown in Algorithm 1.

CDHMM is a three-layered stochastic process. The first level, which is related to the choice of the next state, is the same as in the structure with discrete HMM. Although the second and the third levels are identical to the choice of emission symbol with discrete HMM, the second level of CDHMM includes the choice of the mixture density by mixture coefficient. The selection of the output vector by the Gaussian density is the third and last level. Therefore, the classification and training algorithms have to be altered accordingly, although there are few changes in the classification algorithm i.e., the modified probability densities have to be replaced. The Baum–Welch/Viterbi training algorithms are invoked to obtain the optimized state transition labels and the corresponding estimate of the optimized B matrix value. However, the disadvantage of this model may be attributed to its high computational effort. This aspect is attributed to the evaluation of multiple Gaussian distribution mixture densities which may result into a number of parameters, resulting in instabilities.
**Algorithm 1** Procedure to cluster the data using minimum distance algorithm for training the data set.
1:Consider ‘n’ as number of classes and ‘k’ as feature vectors  2:For the initial sample, prefer the first ‘n’ of ‘k’3:In order to populate ‘n’ classes, assign feature vectors to the nearest sample  4:Give index i|k|=n to nominate x(k) associate to class ‘n’. 5:Estimate the cluster size with s[n] and increase (s[n] = s[n] + 1) and go to Step 36:Retrieve the new centers by averaging the feature vectors in each of the class7:Assign a[m] [n] = 0, here ‘m’ is the component of class ‘n’ and x[m][k] is mth component of x(k)8:For each class ‘n’, assign the averages 9:Get the sum of the component values for every ‘m’ and realize the mean cluster ‘n’.10:If there is no change in class or iteration not completed, then goto Step 7 or Step 3 respectively


**Algorithm 2** Procedure to implement continuous density HMM.
1:Input hidden states(S),Clusters(TN),Windows(tuples)2:Read the sensor data3:Clustering using minimum distance algorithm (see Algorithm 1)4:Obtain Π, A, B5:**for** k = 1,…,N **do**
6: Determine $\Pi_{k}$, When $S_{1}=1$
7: $\Pi_{k}=\frac{\mathrm{Number}\;\mathrm{of}\;\mathrm{Occurance}}{\mathrm{Number}\;\mathrm{of}\;\mathrm{training}\;\mathrm{Observation}}$8: **for** m = 1,…,N **do**
9:  Determine $a_{km}$, When $S_{t}=k\;and\;S_{t+1}=m$
10:  $a_{km}=\frac{\mathrm{Number}\;\mathrm{of}\;\mathrm{Occurance}\;\mathrm{for}\;t(S_{t}=k\;and\;S_{t+1}=m)}{\mathrm{Number}\;\mathrm{of}\;\mathrm{Occurance}\;\mathrm{for}\;t\;(S_{t}=k)}$11: **endfor**
12: **endfor**
13: Compute Mean Vector $\mu_{k}$ and Covariance Matrix $V_{k}$14: $\mu_{k}$=$\frac{1}{N_{k}}\sum_{k=1}^{n}O_{t}$, $O_{t}$ ϵ *k*15: $V_{k}$=$\frac{1}{N_{k}}\sum_{k=1}^{n}{\left(O_{t}-\mu_{k}\right)}^{T}\left(O_{t}-\mu_{k}\right)$, $O_{t}$ ϵ *k*16:**for** m = 1,…,N **do**17: **for** k = 1,…,N **do**18:  Compute A-posterior observation probability density $b_{k}\left(o_{t}\right)$
19:  $b_{k}\left(o_{t}\right)$ = $\frac{1}{{\left(2\pi \right)}^{\mu /2}{\left(v_{m}\right)}^{1/2}}$ exp[ $\frac{-1}{2}$$\left(O_{t}-\mu_{k}\right)$$V_{m}^{-1}$${\left(O_{t}-\mu_{k}\right)}^{T}$] 20:  **endfor**21:  **endfor**22:Compute state optimized likelihood using Viterbi algorithm


### 3.2. Learning Vector Quantization Neural Network

Learning vector quantization (LVQ) is a supervised version of vector quantization developed by Kohonen which fundamentally utilizes labelled input data and is based on a competitive (winner-takes-all) learning strategy. The basic LVQ approach is quite intuitive. It is based on a standard trained self organizing map (SOM) with input vectors x and weights/Voronoi vectors $w_{j}$. The main objective of the LVQ1 network is to find the appropriate output unit that is closest to the input vector. If x is the training vector $\left(x_{1},\ldots \ldots x_{i},\ldots \ldots x_{n}\right)$ and $w_{c}$ is the weight vector for the *j*th output unit $\left(w_{1j},\ldots .w_{ij},\ldots .w_{nj}\right)$ belonging to different classes, then the weights move away from the input vector [45]. For each training input vector x, the *j*th output is obtained so that the Euclidean distance ‖$x-w_{j}$‖ between the input vector and weight vector for the *j*th output unit is minimized. Simultaneously, the weight vector $w_{j}$ for the *j*th output unit is updated.

if

(1) $$T=c_{j},then$$

(2) $$w_{j}\left(new\right)=w_{j}\left(old\right)+\alpha \left[x-w_{j}\left(old\right)\right]$$

if

(3) $$T\neq c_{j},then$$

(4) $$w_{j}\left(new\right)=w_{j}\left(old\right)-\alpha \left[x-w_{j}\left(old\right)\right]$$

T = class for the training vector
*x* = training vector
$w_{j}$ = weight vector
$c_{j}$ = class represented by *j*th output unit

However in LVQ2, learning weights are updated by using two codebook vectors, $m_{i}$ and $m_{j}$, which are taken as the two nearest neighbors of the training vector *x*. This concept does not form a part of the strategy in LVQ1. In this case, $m_{i}$ belongs to correct class and $m_{j}$ belongs to the wrong class. Furthermore, in LVQ2 the vectors $m_{i}$ and $m_{j}$ are updated only when *x* should be located within a zone called a “window” of relative width w if min$\left(\frac{d_{i}}{d_{j}},\frac{d_{j}}{d_{i}}\right)$s, where s=$\frac{1+w}{1-w}$. The complete procedure to implement LVQ2 is given in Algorithm 3.
**Algorithm 3** Procedure to implement learning vector quantization (LVQ-2).
1:Prepare reference vectors, weight vectors $w_{j}$ and learning rate (α)2:**while** (stopping condition false) **do**
3: **for** every training input vector *x* **do** steps 4 to 74:Discover the *j*th output for each training input vector where Euclidean distance ‖$x-w_{j}$‖ should be minimum, renew weight vector $w_{j}$5:If the class defined for training vector is equal to the class represented by *j*th output unit, then $w_{j}\left(new\right)=w_{j}\left(old\right)+\alpha \left[x-w_{j}\left(old\right)\right]$6:else $w_{j}\left(new\right)=w_{j}\left(old\right)-\alpha \left[x-w_{j}\left(old\right)\right]$7:  **endfor**8:  **endWhile**9:Repeat the above procedure for each instance in training dataset by decreasing the learning rate.


### 3.3. Probabilistic Neural Network

**Figure 3:**
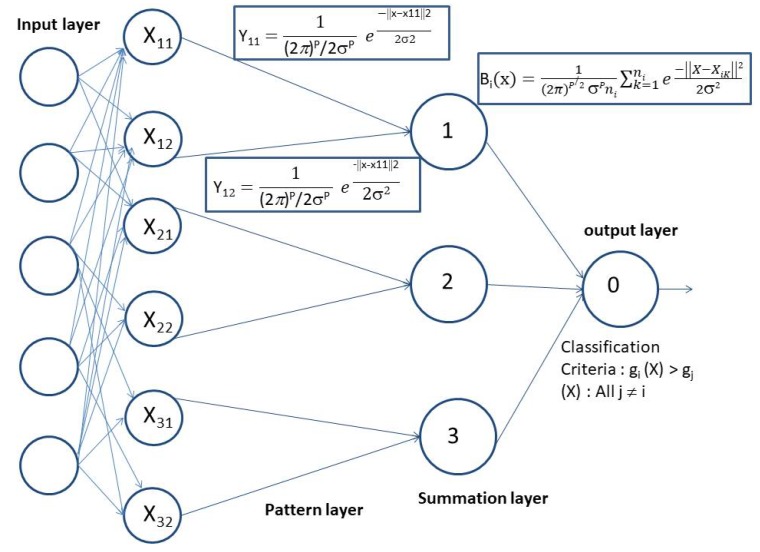
Architecture of probabilistic neural network.

A probabilistic neural network (PNN) is predominantly a classifier which can map any input pattern to carry out multiple classifications of categories. Basically, PNN is an implementation of a statistical algorithm called the kernel discriminant analysis in which the operations are organized using four different layers, namely input layer, pattern layer, summation layer, and output layer [46]. The architecture of the probabilistic neural network is shown in Figure 3. According to the classification theory, if the probability density function (PDF) of each of the populations is known, then an unknown, X, belongs to class “i” if:

(5) $$f_{i}\left(X\right)f_{j}\left(X\right),\forall j\neq i$$

PNN incorporates with Bayesian method for decision-making along with a non-parametric estimator to achieve the probability density function (PDF). Algorithm 4 shows the procedure to implement the PNN.

In a population, the PDF for a single sample is given by

(6) $$\frac{1}{\sigma }w\left(\frac{x-x_{k}}{\sigma }\right)$$

where, *x* = unknown input,
$x_{k}$ = *k*th sample,
w = weighting function,
σ = smoothing parameter.

Probability density function for a single population is given by,

(7) $$\frac{1}{n\sigma }\sum_{k=1}^{n}w\left(\frac{x-x_{k}}{\sigma }\right)$$

When Gaussian function is used as an activation function then the PDF becomes

(8) $$g\left(x\right)=\frac{1}{n\sigma }\sum_{k=1}^{n}e^{-\frac{{\left(x-x_{k}\right)}^{2}}{\sigma^{2}}}$$

PNN is one among a few significant powerful statistical classification techniques which when compared to the multilayer feed forward NN, outperforms the latter due to its rapid training speed and operational behavior of the hidden layer with functionally effective kernel functions. Even though PNN has some minor weaknesses related to the requirement of large memory capability in fast classification, the advantages of the PNN is that it encompasses the capability to train many orders of magnitude faster than the multi-layer feed forward NN, its ability to provide mathematically acceptable confidence levels during discrimination, its built-in strength to handle the effect of outliers etc.
**Algorithm 4** Procedure to implement Probabilistic Neural Network (PNN).
1:For every individual training input vector X(i), where i=1,2,3…… n, repeat steps 2 to 52:Generate pattern unit $P_{i}$3:Assign weight vector to pattern unit $P_{i}$ as  $W_{i}=X\left(i\right)$4:Connect pattern unit to summation unit5:If X(i) matches with class 1, then associate pattern unit $P_{i}$ to summation unit $U_{1}$ else associate to $U_{2}$6:At the pattern layer, enumerate Gaussian function values for every class by using the following equation  $g_{i}\left(x\right)$ = $\frac{1}{\sqrt{2\pi }\sigma^{2}}e^{\frac{-||x-x_{j}{\left|\right|}^{2}}{2\sigma^{2}}}$7:The input vectors that belong to same class is summed in the summation unit  
$f_{i}\left(x\right)=\sum_{i=1}^{n}g_{i}\left(x\right)$
8:With the help of ‘f’ values the output layer makes the decision to assign classes for the input vectors. 9:If $f_{i}\left(x\right)\geq f_{j}\left(x\right)$, then x belong to class 1 else x belong to class 2.


### 3.4. Adaptive Probabilistic Neural Network

It is evident from previous sections that the construction of PNNs for dynamic events is challenging when compared to stationary events. For designing PNNs for the Bayes-optimal decision surfaces (dynamical in nature), it is required to select a smoothing parameter and a learning sequence. This sequence should satisfy conditions that are exemplary for the stochastic approximation scheme [47].

The adaptive probabilistic neural network (APNN) is a basic variant of the probabilistic neural network (PNN). It provides a feasible mechanism to vary the smoothing parameter σ or the variance parameter within a particular class node, while the PNN uses the common variance parameter for all the classes. Based on computing an average distance, σ = g.d${}_{ave}$ is determined by the Euclidean distance, whereby PNN employs different values of σ for each class among feature vectors. It is important to note that “g” now serves as the tuning (free) parameter and can be adjusted to suit appropriate density of datasets. Moreover, APNN uses a simplified formula of PDF which obviates the necessity for normalization, so that a considerable amount of computation is reduced.

### 3.5. Radial Basis Function Neural Network

Radial basis function (RBF) neural network implements a supervised learning strategy and can be realized as single or multilayer networks either in linear or non-linear fashion. If the bias function is used as a non-linear model in RBF network then it can expand its hidden layer by one or more. Usually RBF consists of three layers, namely the input, hidden, and output layer. The input layer consists of $k_{0}$ source nodes, whereas $k_{0}$ is the dimensionality of the input vector X. The hidden layer units are also known as radial centers, which provide a set of functions that constitute an arbitrary basis for the input vectors. Radial centers are represented by the vectors, namely $c_{1},c_{2},\ldots ,c_{h}$. Inside the hidden layer unit, the input vectors are transformed into nonlinear functions whereas the hidden unit vectors are transformed as linear functions to the output layer. In such a case the dimension of each radial center for a ‘n’ input network is n*1. If the input vector $k_{i}$ lies in the respective field then the center would activate $c_{j}$, and by using proper choice of weights its target output is given by

(9) $$y=\Sigma \phi_{j}w_{j},\phi =\phi (\|x-c_{j}\left\|\right)$$

When the Gaussian function is used as the radial bias function then the computational unit present in the hidden layer of the network is given by

(10) $$\phi \left(z\right)=e^{\frac{-z^{2}}{2\sigma^{2}}}$$

σ is a measure of width of the *j*th Gaussian function with center $c_{j}$. The activation function of the hidden unit layer computes the Euclidean distance between the input vector and the center of that unit.

## 4. Fault Detection and Classification Framework

The process of the detection and classification of the sensor faults using CDHMM with NNs is summarized as follows:

1. Raw data, from a sensor node in the wireless sensor network, is pre-processed to remove any anonymous data.
2. The fault injection is carried out on the raw data using the established models for various faults.
3. The fault data is fed as the input for CDHMM with the number of hidden states, the number of clusters and the windows defined initially.
4. The transition matrix from the CDHMM is presented as the input to all the classifiers one at a time.
5. The output of the classifiers are compared and the best overall outcome of the classifier is determined based on an appropriately chosen voting scheme (Byzantine or majority voting strategy).

**Figure 4:**
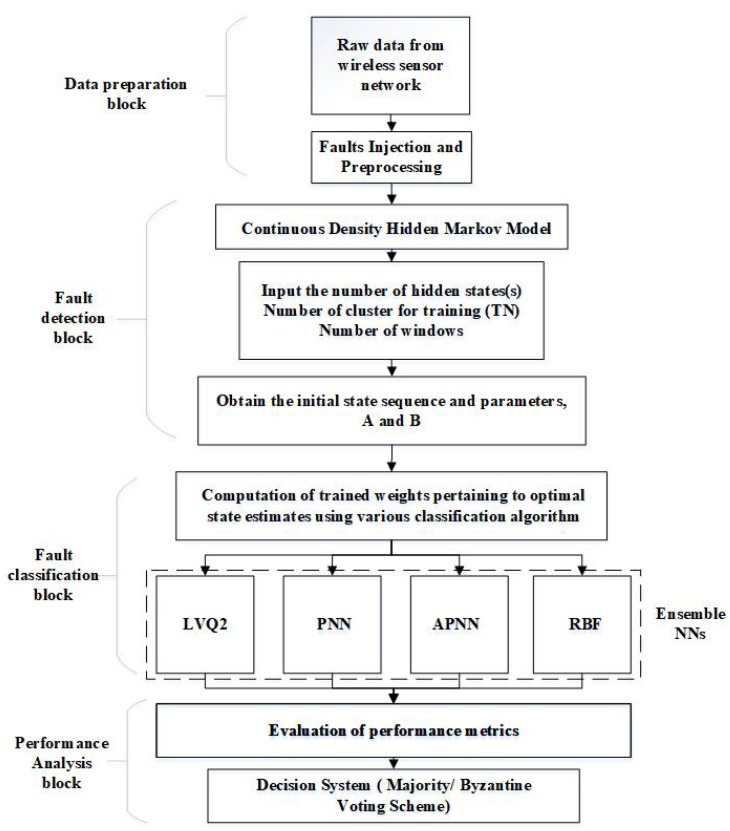
Proposed framework for the detection and classification of sensor faults.

The proposed scheme of hybrid CDHMM with NNs is shown in Figure 4.

## 5. Experimental Results and Analysis

**Figure 5:**
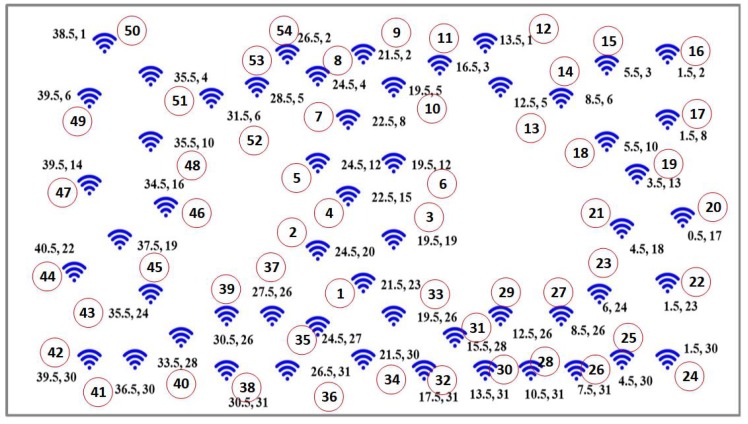
Typical wireless sensor nodes arrangement [48], where wireless sensor nodes are depicted with symbol in ’Blue’ and corresponding node number.

In this section, the evaluation of the proposed hybrid classifiers is carried out on the real time data set provided by Intel Berkeley research Lab [48]. There are 54 nodes installed in the laboratoryin total, recorded from 28th February to 5th April 2004. The acquisition of data had been previously carried out for every 31 seconds from the Mica2Dot sensors along with the topology information. This data had been collected using the TinyDB query processing system which was built on the TinyOS platform. The location of various nodes is shown in Figure 5. The data set contains light, temperature, humidity, and voltage values which had been recorded over the period of a month. In this research, only temperature and light data of one of the nodes (located at (12.5, 26), see Figure 5) was utilized for fault analysis. The reason for choosing, among a large set of parameters, only these two parameters is attributed to the strong correlation between them at a particular instant of time.

The implementation of hybrid CDHMM with NNs adopts a three-level model. The details of the steps that were followed for the evaluation are shown in Figure 4. The primary aim of this research is to detect and classify the time dependent faults that are due to both discrete or continuous types. The bias and drift faults are considered as continuous faults, while the spike and random faults are characterized as discrete faults. The first step of the detection is to prepare the dataset according to the requirements of the algorithm. All the proposed algorithms are realized in MATLAB 9.6. The proposed NNs have been both verified and validated thoroughly before obtaining the appropriate choice of optimal hyper-parameters. In each case, detailed analyses based on raw datasets were taken up and two major validation techniques, the k-fold method and the one-hold-one out method, were performed.

**Figure 6:**
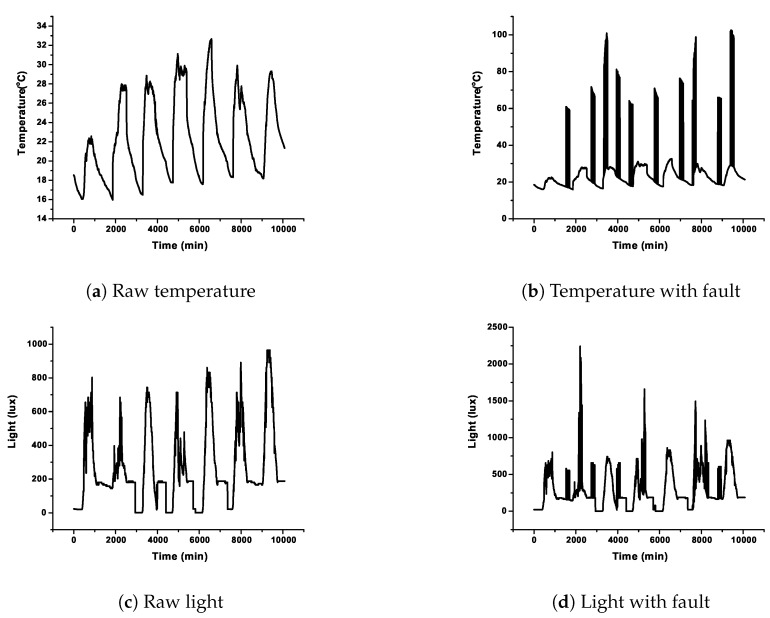
Temperature and light data with and without fault.

In this research, five case studies were taken up for detailed analysis wherein the data sets were organized into two categories, the normal and varying percentage of sensor faults. The dataset was composed of raw light and temperature feature data with an array size of 40 rows by 60 columns. Therefore, a dataset of 2400 data without any fault was prepared. Subsequently, bias, drift, random, and spike fault were introduced on the raw data using the algorithm as discussed in [49]. The methods followed for modeling the fault data were well proven and adopted by various researchers around the globe [50,51]. Figure 6a,c, shows the raw signal of temperature and light. Figure 6b,d, depicts injected random faults in temperature and light. The rate of fault injection started from 10% to 50% with a step size of 10 percent for all the four types of faults. A total of 2400 sets of data were prepared for the performance analysis of the algorithm for each fault type. In each dataset, $\frac{2}{3}$ of the datasets were utilized for training while the rest of the data were taken up for testing. The first level of the model involved obtaining the fault detection state which comprised of CDHMM, and the outputs were state transition matrix “A” and probability density matrix “B”. In the second stage, the optimized values of the B matrix obtained from the Viterbi algorithm were presented as the input to the next stage which comprised of various neural networks to classify the different faults. In each classifier, the kernel was a Gaussian function, resulting in a group of NNs called ensemble classifiers [52]. The performance of the classifiers could be determined using the four evaluation parameters, namely detection accuracy (DA), false positive rate (FPR), F1-Score, and Matthews correlation coefficient (MCC). Finally, based on an appropriately-chosen voting scheme, the best classifier was determined. To ensure credible validation and reliability of the proposed hybrid CDHMM-NN system, the fault detection techniques were executed repeatedly for obtaining consistent results, and average values were considered for analysis.

The detection accuracy (DA) [18] is defined as

(11) $$DA=\frac{\mathrm{Number}\;\mathrm{of}\;\mathrm{faulty}\;\mathrm{observation}\;\mathrm{detected}}{\mathrm{Total}\;\mathrm{number}\;\mathrm{of}\;\mathrm{faulty}\;\mathrm{observation}}$$

The DA should be higher for an excellent classifier and it denotes the accuracy of the detection of the algorithm. The DA for all the NNs with various faults was determined. The false positive rate (FPR) [18] measures the number of non-faulty data detected as faulty with the total number of non-faulty data. It is defined as follows:

(12) $$FPR=\frac{\mathrm{Number}\;\mathrm{of}\;\mathrm{Nonfaulty}\;\mathrm{data}\;\mathrm{detected}\;\mathrm{as}\;\mathrm{faulty}}{\mathrm{Total}\;\mathrm{number}\;\mathrm{of}\;\mathrm{Nonfaulty}\;\mathrm{data}\;}$$

Matthews correlation coefficient (MCC) [18] determines the accuracy of the algorithm and it is defined as below.

(13) $$MCC=\frac{T_{p}\times T_{n}-F_{p}\times F_{n}}{\sqrt{\left(T_{p}+F_{p}\right)\left(T_{p}+F_{n}\right)\left(T_{n}+F_{p}\right)\left(T_{n}+F_{n}\right)}}$$

where True Positive (T${}_{p}$) shows the correctness of detection of number of data that are faulty. True Negative (T${}_{n}$) is defined as the detection of number of non-faulty data as non-faulty. False Positive (F${}_{p}$) is defined as detection of non-faulty as faulty. On the other hand, False negative (F${}_{n}$) is defined as the detection of faulty data as non-faulty. When the MCC is near to + 1, it means the detection is accurate. MCC can be categorized as excellent, moderate and weak based on the values. If MCC is of +0.7 or higher, it can be considered as excellent, from +0.4 to +0.69, it can categorized as good and from +0.30 to +0.39 it is moderate. When it reaches +0.20 to +0.29, it is considered as weak.

The F1-Score [13] is used to measure the performance of the classifier based on false positive and false negative values. It is defined as follows.

(14) $$F1-\mathrm{Score}=\frac{2\times \mathrm{Precision}\times \mathrm{Recall}}{\mathrm{Precision}+\mathrm{Recall}}$$

where, precision = TP/(TP + FP) deals with the accuracy of the detection and recall = TP/(TP + FN) is defined as the measure of determining the particular node as faulty or non-faulty.

### 5.1. Analysis on Capturing the Dynamics of Sensor Faults with CDHMM

**Figure 7:**
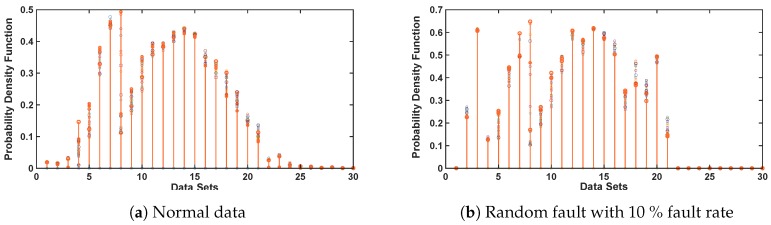
Dynamics of the sensor faults.

Since the major focus of this research is on devising a unique mechanism to ascertain and capture the dynamics of sensor fault by implementing CDHMM, the findings of variation in the state transitions unique to various types of sensor faults are depicted in Figure 7. Here, the transition in the sensor fault is estimated, described and optimized by the probability density function (PDF) of hidden state labels of the CDHMM. Figure 7a shows the PDF for raw data of temperature and light whereas Figure 7b depicts the PDF for random fault with 10% fault rate.

It can be noted clearly that the signature patterns of the optimized B-Matrix for the normal and fault data are distinct. Likewise, for various types or percentage of faults, significant and unique variations of patterns can occur. It is also evident from Figure 7a,b that there are significant changes in the PDF values in the data bin between 0 to 10 and from 15 to 25 bins while the values of B-matrix are very similar in the region of data bins between 10 to 15. This clearly demonstrates the strength of CDHMM as a tool in capturing the dynamics of the time sequence data in wireless sensors. These PDFs are then provided to ensemble classifiers to classify the faulty and non-faulty data.

### 5.2. Detection Accuracy and False Positive Rate Analysis

**Figure 8:**
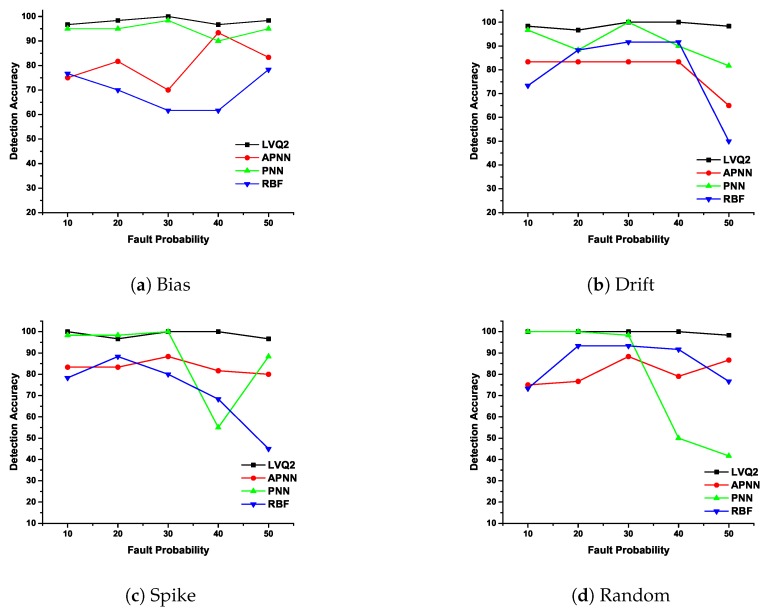
Detection accuracy of various faults with different neural networks (NNs).

**Figure 9:**
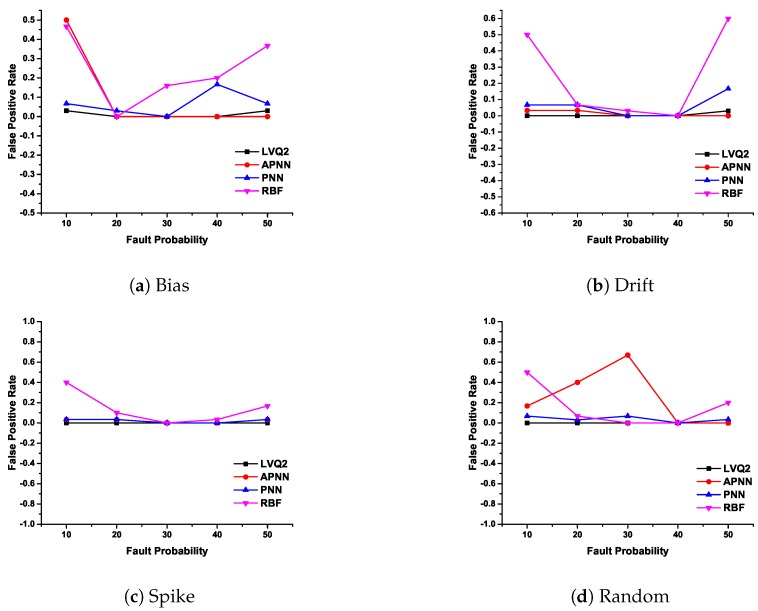
False Positive Rate of various faults with different NNs.

In CDHMM, based on repeated experimentation, iteration value is fixed as 1000. The detection accuracy and false positive rate of various faults are plotted in Figure 8 and Figure 9.

The DA and FPR are determined for different NN classifiers and for different percentage of faults. In Figure 8a, DA is plotted for bias fault and it can be noted that DA for LVQ2 is constant for all percentages of faults whereas APNN showed lower accuracy at smaller fault rates, but performance was better at higher percentages of faults. On the other hand, RBF and PNN did not perform well for higher percentages of faults. In Figure 8b, the LVQ2 and APNN were found to achieve better accuracy compared to RBF and PNN. In Figure 8c, except RBF, all other classifiers demonstrated strong performance. In Figure 8d, for random faults, the LVQ2 and PNN display a high accuracy compared to APNN and RBF. From the aforementioned discussions, it is clear that the DA for LVQ2 was almost constant for all faults compared to other NNs. It is also evident from the analysis that for the lower extent of faults, either LVQ2 or PNN could be preferred. The reasons for the erroneous response of APNN and RBFNN may be attributed to the aspects summarized.

1. Though the APNN comprises a separate variance parameter for each category (class) of defect pattern, it is evinced from this study that the variance parameter displayed a tendency of peaked nature of the Gaussian kernel so as to ensure meaningful separation of hyper-boundaries for classes that are very similar in its patterns.
2. The values of the spread parameter (variance parameter) pertaining to each class resulted in very small values, indicating the nature of the peaked distribution of datasets for each class. This aspect invariably led to overlapped nature of classes and hence arguably higher misclassification rates.
3. On the other hand, the rudimentary version of the RBFNN tried to circumvent the limitations of the backpropagation NN by ensuring learning of weights which then proceeds to obtain a linear separable model to reach the target output. However, the performance of the NN did not indicate considerable improvement in its classification accuracy. This may be attributed to the need for the appropriate choice of centers during the training phase.

The Figure 9a depict the characteristics of LVQ2 for different fault probability and it is considerably strong compared with the other three NNs. The FPR for both PNN and RBF at lower and higher fault rates did not demonstrate satisfactory results. Further, APNN exhibited a poor performance for lower percentages of fault. In Figure 9b, except RBF, all other NNs such as LVQ2, PNN, and APNN detection performed well for all percentage of faults. In Figure 9c, FPR is plotted for spike fault where LVQ2 obtained zero misclassifications for all percentage of faults. This shows the exceptional characteristic of LVQ2 in the precise classification of faults. Incidentally, PNN and APNN exhibit the same variation trend for all probabilities. However, the RBFNN shows a lower accuracy for lesser percentage of faults, and at 30% the performance is almost same as other NNs. However, for higher fault rates, the FPR is within an acceptable limit. In Figure 9d, for random faults, the LVQ2 and PNN are perfectly constant for any percentage of faults, while other NNs eschewed themselves differently for the increase of fault percentage. From these observations, it is clear that the FPR for LVQ2 performance is the best in comparison with other NNs.

The erroneous performance of the RBF may be attributed to the similarity in structure with that of the PNN which also considers a Gaussian-like kernel for estimation of densities and utilizes the variance for describing the distribution of datasets pertaining to the appropriate class. Though the NN is a supervised learning-based architecture, since functionally a similar structure of mathematical formulation and computation is utilized, it is evident that the results of classification accuracy was quite similar to that of the PNN variants considered in this research study.

**Figure 10:**
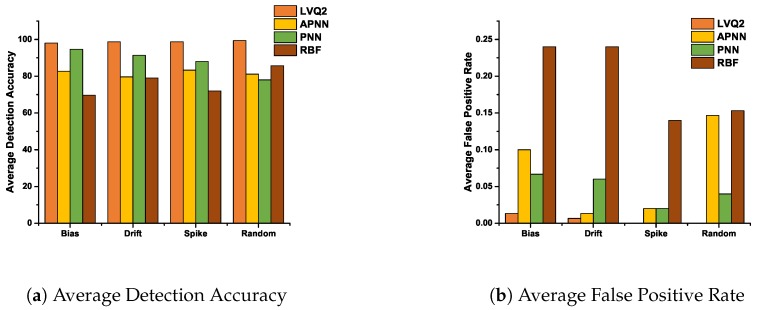
Average detection accuracy and average false positive rate of various NNs for all faults.

The average detection accuracy and false positive rate for various NNs of all faults are plotted in Figure 10. These can be determined by averaging the detection accuracy and the false positive rate for all fault probability for each fault. The performance of various NNs can be easily comprehended from the bar graphs in Figure 10a,b. In Figure 10a, LVQ2 and PNN performed better than RBF and APNN in detecting accurately the faults. On the other hand, detection by APNN is almost constant for all the faults. Similarly, RBF is better for higher fault rates compared to lower cases. From Figure 10b, it is evident that LVQ2 did not detect any non-faculty data as a faulty type i.e., the false detection had almost zero misclassifications for LVQ2. It is also evident that for all transient or instant faults the PNN could be used as an alternate to LVQ2. For constant fault, APNN may be selected in place of LVQ2. Similarly, the performance of RBFNN was observed to be only just average for any type of faults. Table 1 shows the consolidated detection performance of various classification techniques. In terms of ranking, it is summarized that LVQ2 is the most preferred, followed by the PNN, APNN, and RBFNN classifiers.

It is evident that for large values of the smoothing parameters there are more misclassifications, which is obvious due to the nature of the density of the overlapped data because of various types of sensor faults taken up for study. However, with reduced values of smoothing parameters, substantial improvement in classification accuracy is evinced. These aspects are observed in the detailed analysis of both PNN as well as APNN. In the case of APNN with variation in “g”, it is observed that unique smoothing values resulted in better class discrimination. It is also observed that for the best performance of APNN, having more training sets which could provide appropriate class representative vectors would yield enhanced classification rates.

### 5.3. F1-Score and Matthews Correlation Coefficient Analysis

**Figure 11:**
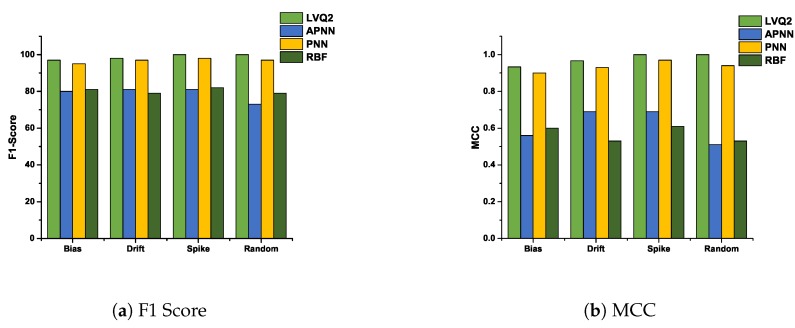
F1-Score and MCC value of various NNs for all faults at 10 % fault rate.

**Figure 12:**
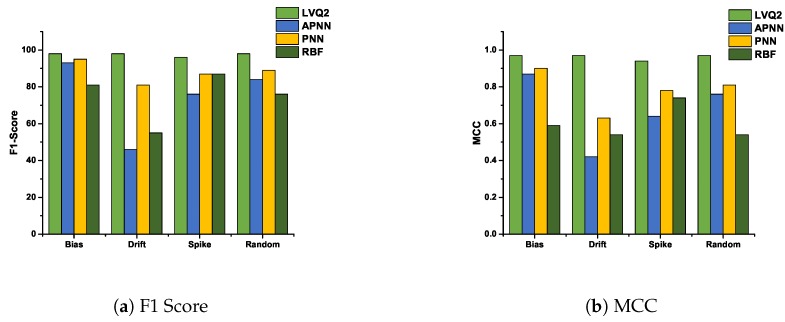
F1-Score and MCC value of various NNs for all faults at 50 % fault rate.

In this subsection, we analyze the F1-Score and Matthews correlation coefficient for various faults at 10% and 50% fault rates. These two fault rates are chosen to analyze the performance of various hybrid CDHMM-NNs during low and high fault occurrence in a sensor node. Figure 11a and Figure 12a shows F1-Score for all NNs at minimum and maximum percentage of faults. The comparison of these two characteristics reveals a similar trend follows for both LVQ2 and PNN. The characteristics are almost constant for all faults and values; also, it lies within the acceptable limit in both percentages of faults. Even the APNN and RBF give better performances and the least percentages of faults. On the contrary, while the percentage of fault increases, APNN performs well for bias and random fault, whereas negative trend in drift fault. On the contrary, RBF performs to a moderate level for bias and spike faults, while for drift and random, the performance does not meet the requisite expectations. Figure 11b and Figure 12b shows MMC values for all NNs at a minimum and maximum percentage of faults. From the comparison, the LVQ2 performance is constant for all faults, and PNN is next to LVQ2 and its trend also lies within the acceptable limit for all percentages of fault. However, APNN performs well for bias and random fault at 50 % fault, while for lower level faults it shows moderate performance. At the same time, RBF performs to an acceptable level for bias and spike faults whereas in drift and random types, the performance did not meet the expectations at both lower and higher percentage of faults. Table 2 shows the performance of all the NNs for all faults at 10% and 50% fault rate. These coefficients are determined by repeated simulation of fault data for various algorithms. In this research, the LVQ2 shows an excellent performance compared to all other NNs. Furthermore, the performance of PNN is par with LVQ2 at lower percentage of faults.

Though the LVQ2 does not directly utilize the Gaussian kernel, the inherent structure of obtaining the distance vector through the operator ϵ in LVQ2 provides a window (range) for the association of similar classes to particular classes during training. Hence, structurally similar types of NNs have been taken up in this research for carrying out meaningful and cogent analyses. For lower values of ϵ the density of data with a peaked variance is observed. The tendency of data displayed a peaked distribution for ϵ. When there is substantial overlap in the signal (fully or partially overlapped), this may be attributed to the dynamic nature of normal or abnormal fault analyses.

Normally, in order to improve the classification accuracy, the number of iterations in the algorithm is increased. However, this would not be a convincing strategy for the improvement of detection. This is attributed to the fact that the traditional mean square error-based algorithms may not result in global minima during the training phase. Therefore, in our research, the iteration values have been optimized to be 1000 throughout the analysis of all fault classifications.

### 5.4. Ensemble Classifier-Based Majority Voting Decision

In this subsection, the research analysis has been consolidated to ascertain the classification accuracy of various CDHMM-NN, and to determine the classification capability of an ensemble classifier for different percentage of faults using the majority and Byzantine voting systems which are appropriate for the hybrid CDHMM-NNs taken up for detailed studies. The total training and testing datasets considered for each percentage of fault is 60. For HMM-RBF, the tuning parameters α and η are fixed as 0.06 and 0.06 respectively. In HMM-PNN, σ is taken as 0.1, while in HMM-APNN, g is taken as 0.9 whereas in HMM-PNN, σ is fixed as 0.6. It is worth mentioning that these values are optimized after multiple iterations, and they are fixed as constant for all percentage of faults. Based on detailed analyses carried out on gamut of faults encountered in sensor input datasets, few major findings are summarized:

1. It is evident from Table 3, that for all versions of CDHMM-NN classifier systems that with increasing percentage of faults in the dataset, better classification accuracy of the hybrid classifiers have been observed. This aspect is obvious since unique features that describe the nature of faults are exhibited in the case of higher density estimates of such datasets. Though in this study and implementation a common value of the tunable parts of the NN (variance parameter, ϵ, η) has been utilized for assessing the role played by the Gaussian kernel in creating hyper-boundaries during the training phase, appropriate tweaking of the free parameter led to much more enhanced results than the outcome indicated in Table 3.
2. Though for the dataset taken up during the training phase in this research work resulted into superior classification capability of LVQ2 as compared to other NNs, it is to be noted that for large real-time datasets (with substantially varying background noise) the decision based on only LVQ2 will not be the best strategy as more training, input validation studies etc. would be essential before arriving at a meaningful decision. It clearly indicates that the decision based on LVQ2 as a single NN alone would be insufficient for obtaining a comprehensive decision.
3. Another interesting aspect is on the role played by the Gaussian kernel utilized as the common underlying activation function in the ensemble classifier system. It is observed that similar types of misclassifications have been reported during detailed studies based on various faults, and the nature of data distribution and the density function resulted in the sharp nature of Gaussian distribution functions for each class. This aspect is made evident from the few significant changes in the misclassification of a few of the input datasets.
4. The ensemble classifier performed considerably well in the case of the hybrid CDHMM-NN classifier system which utilizes the majority voting scheme, while the Byzantine voting scheme which utilizes a more conservative procedure for decision-making displayed inferior results.

## 6. Conclusions

A hybrid CDHMM with NN was implemented to detect and classify the faults such as bias, random, drift, and spike. The dataset consisted of temperature and light, while pre-processing and injection of fault from 10% to 50% was carried out before it was presented to the CDHMM. The probability density function from CDHMM was given as input for training and testing data to carefully chosen classifiers, namely LVQ2, PNN, APNN, and RBFNN. The performance of various classifiers were compared in terms of detection accuracy, false positive rate, F1-score and Matthews Correlation Coefficient. The results showed that the hybridized CDHMM with LVQ2 turned out to be the best machine learning algorithm for determining the instant changes in the sensor data due to the effect of various faults. Although LVQ2 performed well for most of the faults, the other NNs showed better performance for unique and specific faults. Therefore, the results showcased in this work were most appropriate for the selection of NN for hybridization with CDHMM for a particular fault. Hence, the proposed method is best suited for a time-critical wireless sensor network, where the detection of faults is essential instantly, yet with substantial reliability. Further, this research work reports detailed studies based on only one major kernel, i.e., Gaussian kernel. Although the Gaussian kernel is the most common and popularly utilized kernel function when the nature of distribution of data is random, other distribution functions with kernels such as elliptical, Laplacian, Cauchy etc. could provide better representation of the data. These hybridized CDHMM and NNs can be extended to a few possible aspects in WSN sensor fault detection:

1. Prognosis and prediction of remaining useful life and ageing characterization of the sensors could be possibly attempted using HMM and other methods such as bath-tub distribution functions, Weibull distribution, and other higher order statistical measures.
2. Online detection of faults can be implemented with this hybridized algorithm in a real-time environment. However, challenges related to training speed in utilizing the HMM may be attempted by taking up alternative algorithms (for maximum likelihood estimation and optimization of transition states).
3. From the perspective of the training and implementation of more parsimonious sets of centers for NN training, advanced algorithms that optimize and provide reasonably good representative centers that ensure compact training sets may aid one in reducing the time during the training phase, specifically in on-line fault detection.

## Figures and Tables

**Table 1:** Detection performance of various classification techniques.

Techniques	Average DA	Average FPR
LVQ2	98.66 %	0.005
APNN	81.69 %	0.07
PNN	87.99 %	0.047
RBF	76.58 %	0.193

**Table 2:** MCC of various classification techniques at 10 % and 50% fault rate.

Techniques	MCC(10%)	MCC(50%)	Performance
LVQ2	0.95	0.96	Excellent
APNN	0.61	0.71	Good
PNN	0.93	0.78	Good
RBF	0.57	0.52	Moderate

**Table 3:** Classification accuracy of hybrid continuous density hidden Markov model (CDHMM)-NN ensemble classifier system with different voting schemes.

Type of Dataset	Classification Accuracy of CDHMM-NN Classifier System (%)				Accuracy of Ensemble Classifier	
	HMM-LVQ2	HMM-APNN	HMM-PNN	HMM-RBF	M.Voting(%)	B.Voting(%)
**Bias Fault**						
10%	96.67	75	95	76.67	95	76.67
20%	98.33	81.67	95	70	95	81.67
30%	100	70	98.33	61.67	98.33	70
40%	96.66	93.33	90	61.67	93.33	90
50%	98.33	83.33	95	78.33	95	83.33
**Drift Fault**						
10%	98.33	83	96.67	73.33	96.67	83
20%	96.67	83.33	88.33	88.33	88.33	83.33
30%	100	83.33	100	91.67	100	91.67
40%	100	83.33	90	91.67	91.67	90
50%	98.33	65	81.67	50	81.67	65
**Spike Fault**						
10%	100	83	98.33	78.33	98.33	83
20%	96.67	83.33	98.33	88.33	98.33	88.33
30%	100	88.33	100	80	100	88.33
40%	100	81.67	55	68.33	81.67	68.33
50%	96.67	80	88.33	45	88.33	80
**Random Fault**						
10%	100	75	100	73.33	100	75
20%	100	76.67	100	93.33	100	93.33
30%	100	88.33	98.33	80	98.33	88.33
40%	100	79	50	91.67	91.67	79
50%	98.33	86.67	41.67	76.67	86.67	76.67
